# *In vitro* and *in vivo* antibacterial activity assays of carvacrol: A candidate for development of innovative treatments against KPC-producing *Klebsiella pneumoniae*

**DOI:** 10.1371/journal.pone.0246003

**Published:** 2021-02-22

**Authors:** Gleyce Hellen de Almeida de Souza, Joyce Alencar dos Santos Radai, Marcia Soares Mattos Vaz, Kesia Esther da Silva, Thiago Leite Fraga, Leticia Spanivello Barbosa, Simone Simionatto

**Affiliations:** 1 Laboratório de Pesquisa em Ciências da Saúde, Universidade Federal da Grande Dourados—UFGD, Dourados, Mato Grosso do Sul, Brazil; 2 Centro Universitário da Grande Dourados–UNIGRAN, Dourados, Mato Grosso do Sul, Brazil; Universita degli Studi della Campania Luigi Vanvitelli, ITALY

## Abstract

Dissemination of carbapenem-resistant *Klebsiella pneumoniae* poses a threat to the successful treatment of bacterial diseases and increases the need for new antibacterial agents development. The objective of this study was to determine the antimicrobial activity of carvacrol against multidrug-resistant *K*. *pneumoniae*. Carbapenemase production was detected by MALDI-TOF. The PCR and sequencing showed that the *bla*_KPC-2,_ *bla*_OXA-48_, *bla*_NDM-1_, *bla*_CTX-M-8_ genes were present in carbapenem-resistant *K*. *pneumoniae* strains. The polymyxin-resistant *K*. *pneumoniae* strain exhibited alterations in *mgr*B gene. The antimicrobial activity of carvacrol was evaluated *in vitro* using broth microdilution and time-kill methods. For this, carbapenem-resistant *K*. *pneumoniae* and polymyxin-resistant strains, were evaluated. The *in vitro* results showed that carvacrol had antimicrobial activity against all isolates evaluated. The survival curves showed that carvacrol eradicated all of the bacterial cells within 4 h. The antimicrobial effect of carvacrol *in vivo* was determined using a mouse model of infection with *Klebsiella pneumoniae* carbapenemase (KPC). The treatment with carvacrol was associated with increased survival, and significantly reduced bacterial load in peritoneal lavage. In addition, groups treated with carvacrol, had a significant reduction in the total numbers of white cell and significantly increased of platelets when compared to the untreated group. *In vivo* and *in vitro* studies showed that carvacrol regimens exhibited significant antimicrobial activity against KPC-producing *K*. *pneumoniae*, making it an interesting candidate for development of alternative treatments.

## Introduction

Multidrug-resistant (MDR) infections are considered a major public health problem [1, 2]. The emergence of MDR bacteria and the lack of new antibiotics is a worrying prospect [3]. A recent report suggests that failing to control drug-resistant infections may cause an excess of 10 million deaths per year and cost up to US$100 trillion by 2050 [4]. Carbapenem-resistant *Klebsiella pneumoniae* strains are frequent cause of healthcare-associated infections and hospital-associated outbreaks [5]. Carbapenem resistance in these pathogens is one of the main causes of morbidity and mortality, and represents a serious health problem worldwide, since it limits therapeutic options for treating infections [1, 6]. Thus, the global spread of MDR has resulted in increased use of polymyxin with the inevitable risk of emerging resistance [7].

According to the World Health Organization (WHO), the control of the spread of antibiotic resistance remain a priority, as well as, development of new therapies against these bacteria [8]. Therefore, the increase in antibiotic-resistant bacteria has revived interest in the study of plant materials as sources of new compounds as alternative therapeutic agents to control pathogenic microorganisms [9, 10]. A major group of plant antimicrobial compounds is represented by essential oils, which consist of complex mixtures of volatile secondary metabolites [11]. Therefore, bioactive compounds extracted from essential oils are promising antimicrobials [12].

The phenolic monoterpene carvacrol [2-Methyl-5-(1-methylethyl) phenol, isomeric with thymol] is a essential component of the essential oils of plants of the Labiatae family, including *Origanum* and *Thymus* and has emerged for its wide spectrum of activity [10]. Some studies have reported their pharmacological activities, such as anti-inflammatory effects, antioxidant, antitumor, analgesic, anti-hepatotoxic, insecticidal and antimicrobial properties [9, 13–17]. This study evaluated the antimicrobial potential of carvacrol *in vitro* and *in vivo* against multidrug-resistant *K*. *pneumoniae* strains.

## Material and methods

### Chemicals

Carvacrol (2-methyl-5-[1-methylethyl] phenol); lot: W224502, purity ≥ 98%) were purchased from Sigma (St. Louis, USA). Tween 80 (0.5%) was used as the solvent for the carvacrol.

### Bacterial strains

Multidrug-resistant *K*. *pneumoniae* strains were obtained from urine culture of hospitalized patients in a tertiary teaching hospital. Bacteria were grown overnight in Mueller Hinton (MH) broth and submitted to phenotypic and molecular assay as previously described [18, 19]. This study was conducted with the approval of the Research Ethics Committee from the Universidade Federal da Grande Dourados (no. 877.292/2014 and 4.014.325/2020). This ethics protocol contemplates informed consent from the patients from whom the strains were isolated.

### Bacterial identification and phenotypic assays

Bacterial species were identified using the Phoenix 100® automated system (BD Diagnostic Systems) and confirmed by matrix-assisted laser desorption/ionization-time-of-flight mass spectrometry (MALDI-TOF) using the Microflex LT spectrometer (Bruker Daltonics, Massachusetts, USA). The minimal inhibitory concentrations (MICs) were determined by broth microdilution according to Clinical and Laboratory Standards Institute (CLSI) standards [20]. Preliminary screening for the presence of carbapenemases was performed by the modified Hodge test (MHT) according to CLSI guidelines. Positive results were confirmed by ertapenem hydrolysis using mass spectrometry [21].

### PCR amplification of resistance genes

The presence of resistance genes (*bla*_TEM-like_, *bla*_SHV-like_, *bla*_CTX-M-1-like_, *bla*_CTX–M-2-like_, *bla*_CTX-M-8-like_, *bla*_CTX-M-14-like_, *bla*_GES-like_, *bla*_KPC-like_, *bla*_SME-like_, *bla*_NDM-1-like_, *bla*_IMP-like_, *bla*_SPM-like_, *bla*_VIM-like_, *bla*_SIM-like_, *bla*_GIM-like_, *bla*_OXA-48-like,_ *mgr*B and *mcr-1*) was evaluated using Polymerase Chain Reaction (PCR) amplification, followed by sequencing as previously described [18, 19].

### Antibacterial activity of carvacrol

Multidrug-resistant *K*. *pneumoniae* strain bacteria were grown overnight in 3 mL Mueller–Hinton (MH) broth at 37°C with constant shaking at 200 rpm. Optical density was measured at 600 nm on the following day, and the cultures were then diluted to ∼5.0 × 10^5^ CFU/mL in a low-binding 96 well microtiter plate containing increasing concentrations of carvacrol (72–0.03 mg/mL). The microtiter plates were incubated at 37°C and the Minimum Inhibitory Concentrations (MIC) and Minimum Bactericidal Concentrations (MBC) of carvacrol were determined as previously described [20, 22]. Polymyxin B (4 mg/L) and amikacin (16 mg/L) (Sigma-Aldrich) were used as controls for the assays with carbapenemase-producing and polymyxin-resistant *K*. *pneumoniae* strains, respectively. Polymyxin B and meropenem sensitive control (*Escherichia coli* 25922) was used as a control to validate antimicrobial susceptibility tests.

### Time-kill test

The time-kill kinetics of the carvacrol at 1 × MBC was performed using the broth macrodilution (MH broth) technique following CLSI guidelines [20, 22]. Time-kill assays were performed using a final inoculum concentration of approximately 5.0 × 10^5^ CFU/mL [23] incubated at 37°C. Samples were collected at 0, 4, 8, 12, 24 h and 100 μL of inoculum was spread out on to MacConkey agar plates. The plates were incubated for 24 h at 37°C and viable cell counts were performed by inspection of colony-forming units (CFUs) to determine the inhibitory effects of carvacrol. The values of the bacterial counts were transformed into CFU/mL and expressed in log to ensure normal data distribution [24]. To confirm the absence of antimicrobial activity of solvent, the negative (water, culture medium and 0.5% Tween 80) and positive (water, culture medium, 0.5% Tween 80 and bacterial suspension) controls were assessed.

### Animals

Seventy-eight female *Swiss* mice *(Mus musculus)*, 8–10 weeks old, weighing approximately 20–30 g (n = 6 in each group) were obtained from the Central Animal Facility of the Universidade Federal da Grande Dourados. Forty-two were used in the lethal dose test and thirty-six in the antibacterial activity assay of carvacrol *in vivo*. The mice were maintained in polypropylene boxes with beddings of wood shaving and provided with commercial feed (Nutival®) and filtered water *ad libitum* throughout the experiment. Light and temperature were controlled using a 12 h photoperiod (12:12 h DL) at 22 ± 2°C and 55 ± 10% humidity on a ventilated shelf (ALESCO®, Monte Mor, Brazil). All the animal care or handling out following the recommendations in the Guide National Council to Control Animal Experimentation (CONCEA). In this study, we assessment of survival, lethal dose, and longevity of infected animals, for that reason humane endpoints were not used, but all efforts were made to minimize suffering. The experiment was only maintained for 24 hours, and the behavior was monitored every hour. After evaluating the infection survival curve, animals that remained alive were euthanized after 24 hours of experimentation. The study was conducted with the approval of the Research Ethics Committee on Animal Use of the Universidade Federal da Grande Dourados (no. 010/2017). The institutional animal ethics committee reviewed and specifically approved the mortality predicted in the study design.

### Lethal dose test

The lethal dose (LD100) and mean lethal dose (LD50) of KPC-producing *K*. *pneumoniae* in mice was assessed as previous described [25]. The mice were injected with a 0.1 mL intraperitoneal aliquot of the following concentrations of KPC-producing *K*. *pneumoniae*: 1.5 ×10^8^, 3.0 ×10^8^, 4.0 ×10^8^, 4.8 ×10^8^, 6.0 ×10^8^ and 9.0 ×10^8^ CFU/mL. The animals were observed for 24 h, the numbers of dead mice in each group were counted and the percentage mortality was calculated. The acute toxicity of carvacrol in mice has previously been described and concentration below of 250 mg/kg showed no mortality in mice [26].

### *In vivo* antibacterial activity

**Fig 1:**
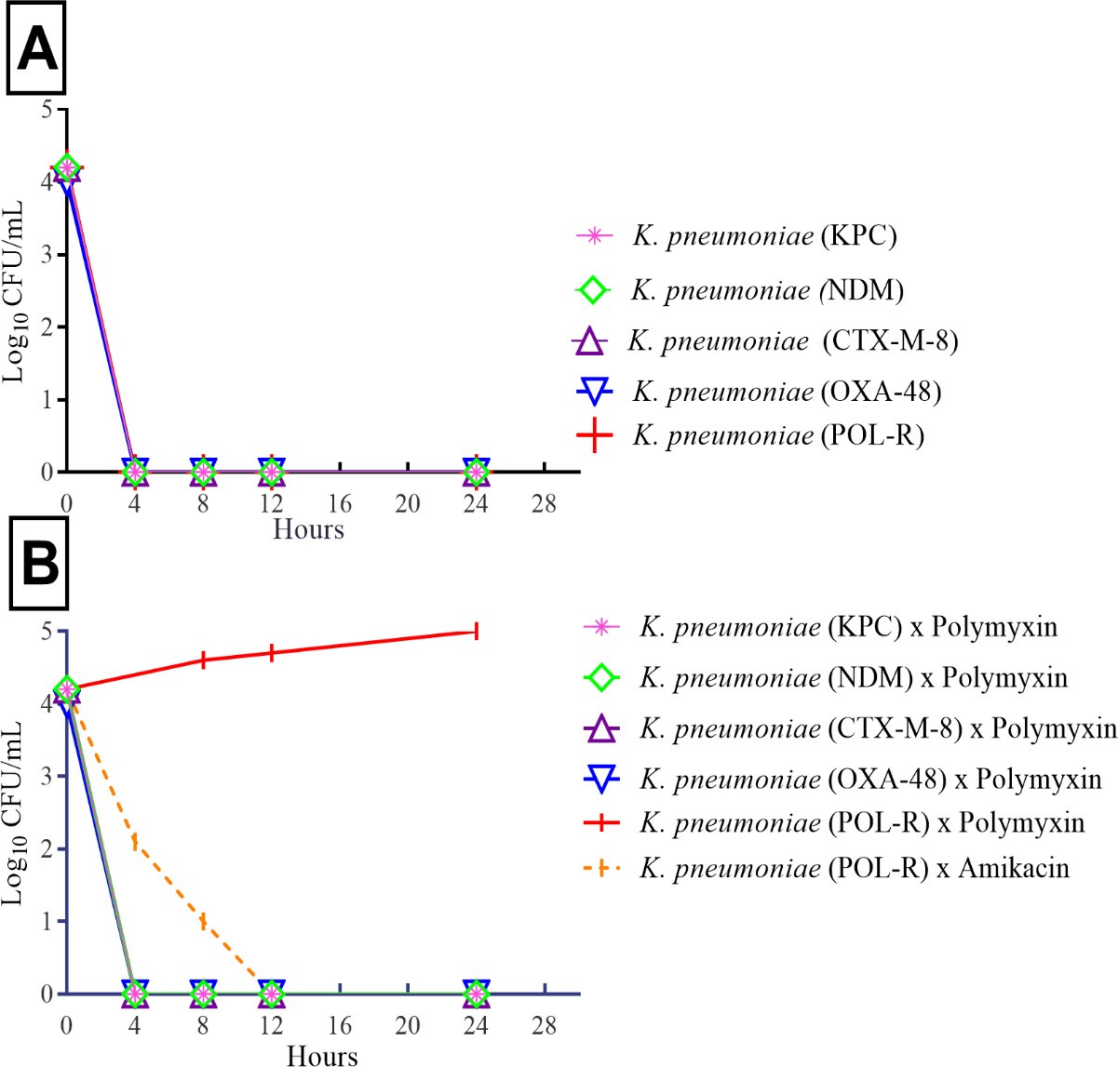
Time-kill curves of multidrug-resistant *K*. *pneumoniae* strains. A) Carvacrol activity against carbapenem-resistant (KPC, NDM, CTX-M-8 and OXA-48) and polymyxin-resistant *K*. *pneumoniae* strains (POL-R). B) Carbapenem-resistant *K*. *pneumoniae* strains (KPC, NDM, CTX-M-8 and OXA-48) tested against polymyxin B and polymyxin-resistant *K*. *pneumoniae* strain (POL-R) tested against polymyxin B and amikacin.

To evaluate carvacrol’s *in vivo* activity, a murine infection model induced by KPC-producing *K*. *pneumoniae* (Fig 1) was performed, as previously described [25], with the following modifications. In brief, female *Swiss* mice were randomly divided into treatment groups (n = 6). All animals were injected with a 0.1 ml intraperitoneal (i.p.) aliquot of 4.0 × 10^8^ CFU/mL (LD_50_). Six groups of 6 mice were treated with the following regimens: polymyxin B (2 mg/kg, intraperitoneal (i.p.), 12/12 h), carvacrol (50 mg/kg, oral gavage (o.g.), 8/8 h), carvacrol (25 mg/kg, o.g., 8/8 h), carvacrol (10 mg/kg, o.g., 8/8 h), infected control group (untreated) and a naïve group. Length of survival was observed in mice surviving at 24 h. All surviving animals were anesthetized with a combination of xylazine, and ketamine (10 and 60 mg/kg, i.p., respectively). Animals were euthanized by exsanguination and organs were collected for analysis. Blood samples were collected for hematological studies using an automated hematology analyzer (Sysmex XE-3000 Hematology Analyzer, Sysmex, Japan). The white cell count (WBC) and platelet abundance (PLT) were determined [27]. Peritoneal fluid samples were obtained through incision and lavage with Milli-Q water by an aseptic technique. Peritoneal lavage fluid was incubated on agar Mueller-Hinton medium supplemented with meropenem (4 mg/L) to verify the presence of KPC-producing *K*. *pneumoniae* and for quantitative cultures, respectively.

After collecting blood, the organs (spleen, liver, lung and kidney) were collected, and weighed. The tissues were buffered formalin-fixed, embedded in paraffin and, sectioned at 5 μm. The sections were stained with hematoxylin and eosin and observed by light microscopy for histopathological evaluation.

### Statistical analysis

Means ± the standard error of the mean (SEM) were calculated and ANOVA/Newman-Keuls post-hoc tests were performed using GraphPad Prism software (version 6.01; Graph-Pad Software Inc., San Diego, CA, USA). Results with P value < 0.05 was considered significant.

## Results

Four clinical carbapenem-resistant *K*. *pneumoniae* strains, and one polymyxin-resistant were included in this study (biorepository accession numbers: KP01, KP02, KP03, KP04 and KP05). Strains showed resistance to the antibiotics tested by broth microdilution as follows: meropenem (MIC >32 mg/L), imipenem (MIC >32 mg/L), ertapenem (MIC >32 mg/L) (Table 1). Carbapenemase production was detected by MHT and MALDI-TOF. PCR amplification and sequencing showed that *bla*_KPC-2_, *bla*_OXA-48_, *bla*_NDM-1_, *bla*_CTX-M-8_ genes were present in carbapenem-resistance *K*. *pneumoniae* strains. The polymyxin-resistance *K*. *pneumoniae* exhibited alterations in the *mgr*B coding sequence. The other genes evaluated (*bla*_TEM-like_, *bla*_SHV-like_, *bla*_CTX-M-1-like_, *bla*_CTX–M-2-like_, *bla*_CTX-M-14-like_, *bla*_GES-like_, *bla*_SME-like_, *bla*_IMP-like_, *bla*_SPM-like_, *bla*_VIM-like_, *bla*_SIM-like_, *bla*_GIM-like_ and *mcr-1*) were not detected in these strains.

**Table 1:** Antimicrobial susceptibility patterns for resistant *K*. *pneumoniae*.

Strains	Genes	MIC (mg/L)			MIC and MBC (mg/L)
		Carbap	Pol	Ami	Carv
KP01	*bla*_KPC-2_	>32 (R)	< 2 (S)	<8 (S)	130
KP02	*bla*_OXA-48_	>32 (R)	< 2 (S)	<8 (S)	130
KP03	*bla*_NDM-1_	>32 (R)	< 2 (S)	<8 (S)	260
KP04	*bla*_CTX-M-8_	>32 (R)	< 2 (S)	<8 (S)	130
KP05	altered *mgr*B	>32 (R)	8 (R)	<8 (S)	130

S: susceptibility; R: resistance; Carbap: meropenem, imipenem and ertapenem; Pol: polymyxin B; Carv: carvacrol; Ami: amikacin.

Carvacrol exhibited significant inhibitory effects, with MICs/MBCs of 130 mg/L for CTX-M-8, OXA-48, KPC, and polymyxin-resistant *K*. *pneumoniae* strains. For NDM-1 producing *K*. *pneumoniae*, the MICs/MBCs were 260 mg/L. MIC and MBC values were equal in the strains evaluated. No inhibitory effects were observed in the positive control, with 0.5% of Tween 80. The survival curves of the strains among 0 and 4th hour suggest a linear drop in viable cell counts (Fig 1A). All strains treated with carvacrol showed decreases in cell counts of approximately two log_10_ CFU/mL. Considering the time of cell death, the results showed total inhibition of carbapenemases-producing *K*. *pneumoniae* strains after 4 h of treatment with carvacrol. Polymyxin B (4 mg/L) was used as a positive control and successfully inhibited the carbapenemase-producing strains within 4 h (Fig 1B). Amikacin (16 mg/L) was used as a positive control and successfully inhibited the polymyxin-resistant strain within 12 h.

**Fig 2:**
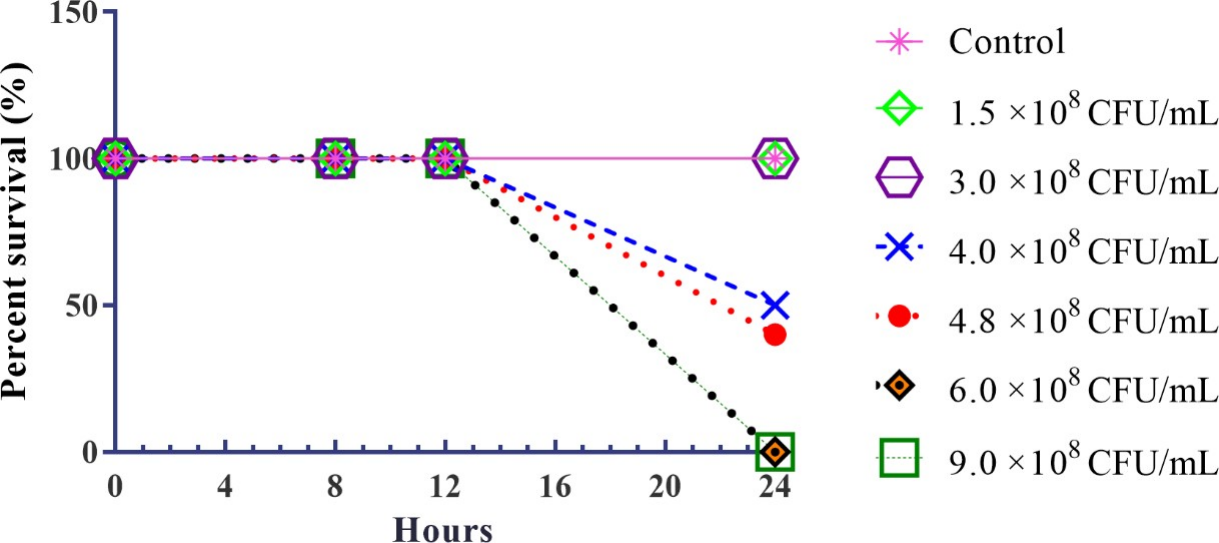
Survival curves mice model infected with different concentrations of KPC-producing *K*. *pneumoniae*. Control: untreated group; concentrations of KPC-producing *K*. *pneumoniae* inoculum (1.5 ×10^8^; 3.0 × 10^8^; 4.0 × 10^8^, 4.8 × 10^8^, 6.0 × 10^8^ and 9.0 × 10^8^ CFU/mL).

KPC-producing *K*. *pneumoniae* strain was selected for the infection in animals. A dose-dependent survival curve was generated using female *Swiss* mice that received intraperitoneal injections of KPC-producing *K*. *pneumoniae* in different concentrations. To determine the LD_100_ and LD_50_ of KPC-producing *K*. *pneumoniae*, percent survival was observed for 24 h after infection (Fig 2). All animals in the control group (untreated) and in the groups infected with concentrations of 1.5 ×10^8^ and 3.0 ×10^8^ CFU/mL of KPC-producing *K*. *pneumoniae*, survived for 24 h. Concentrations of 4.0 ×10^8^ and 4.8 ×10^8^ CFU/mL promoted 50% (LD_50_) and 60% mortality, respectively. All animals infected with concentrations of 6.0 ×10^8^ and 9.0 ×10^8^ CFU/mL died in 24 h (LD_100_).

**Fig 3:**
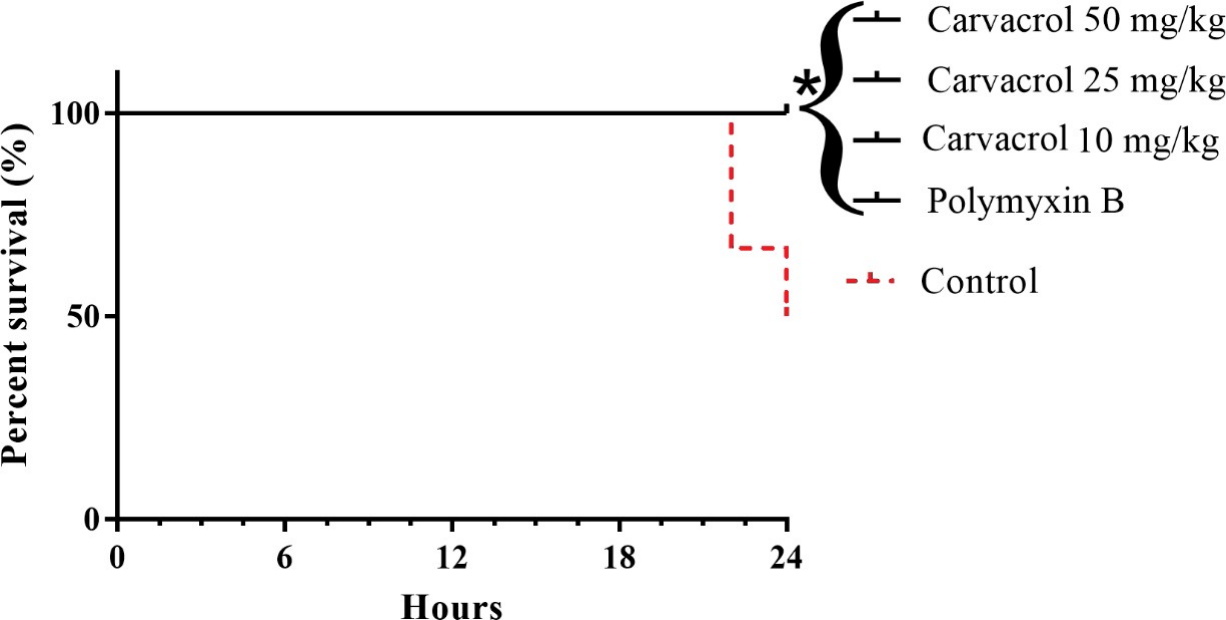
Survival curves of mice infected with KPC-producing *K*. *pneumoniae* and treated with carvacrol. Polymyxin B and untreated (Control) were used as a positive and negative controls, respectively. *P<0.05 compared with the control group.

The antimicrobial activity of carvacrol *in vivo* was evaluated for 24 h, using a model of infection with by KPC-producing *K*. *pneumoniae*. Half of the control group (untreated) died within 24 h after infection (50% mortality). However, all mice of the group treated with polymyxin B (2 mg/kg), and carvacrol (10, 25 and 50 mg/kg) remained alive (0% mortality) (Fig 3).

**Fig 4:**
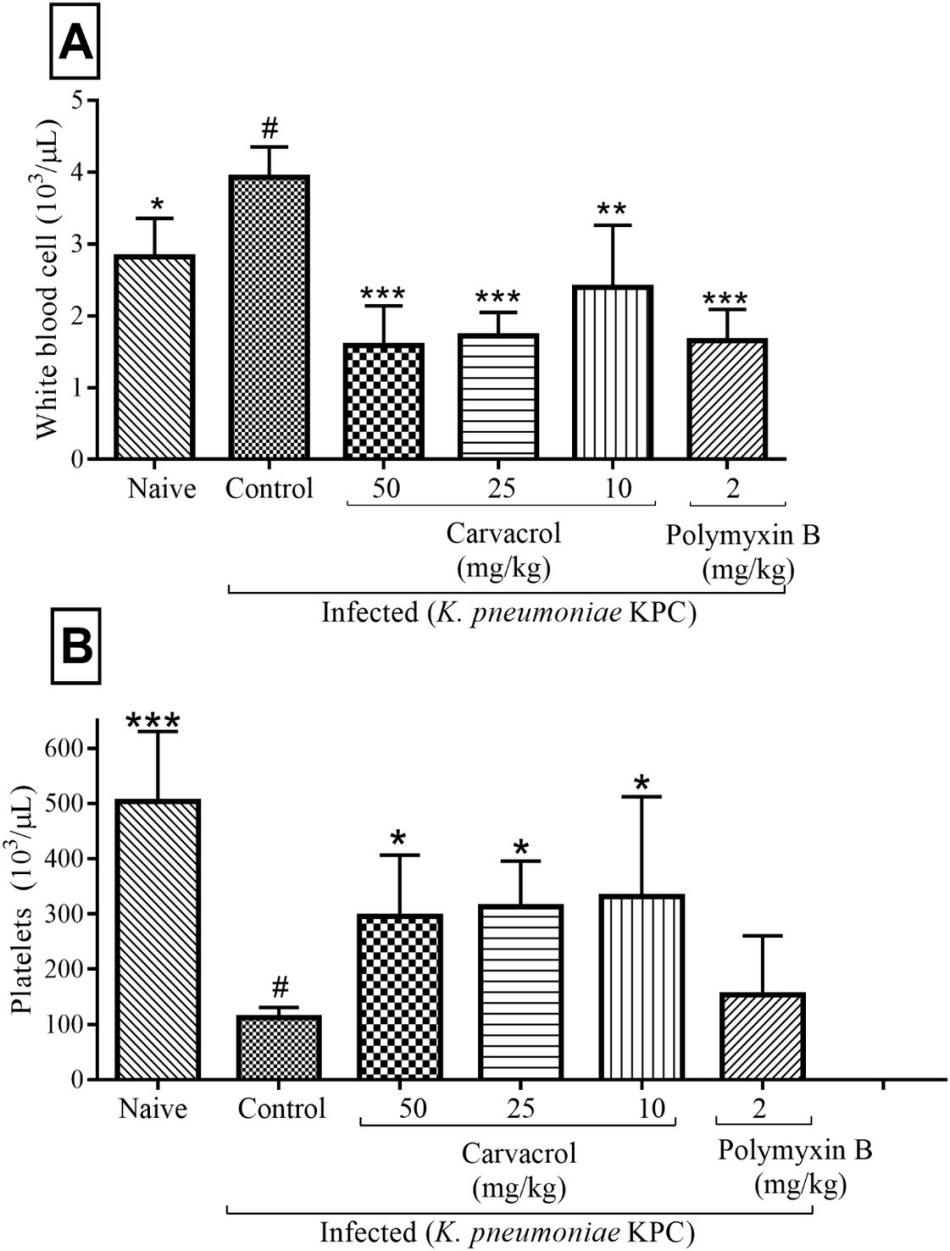
Effects of carvacrol on hematological parameters in mice infected with KPC-producing *K*. *pneumoniae* after 24 hours. WBC (A) and platelets (B). ***P<0.001, **P<0.01 and *P<0.05 compared with the control group (#). Differences among the groups were analyzed by one-way ANOVA followed by the Newman-Keuls test.

In order to characterize the immune response of mice infected and treated with carvacrol, white cell count (WBC) and platelet were determined. Statistical analysis revealed that the white series of cells demonstrated significant alterations. Groups treated with carvacrol (10, 25 and 50 mg/kg) and polymyxin B (2 mg/kg) showed significant reductions in the number of WBC (p < 0.01) (Fig 4A). All groups treated with carvacrol (10, 25 and 50 mg/kg) showed significantly increased platelet counts (p < 0.05), a result not observed in the group treated with polymyxin B (Fig 4B).

**Fig 5:**
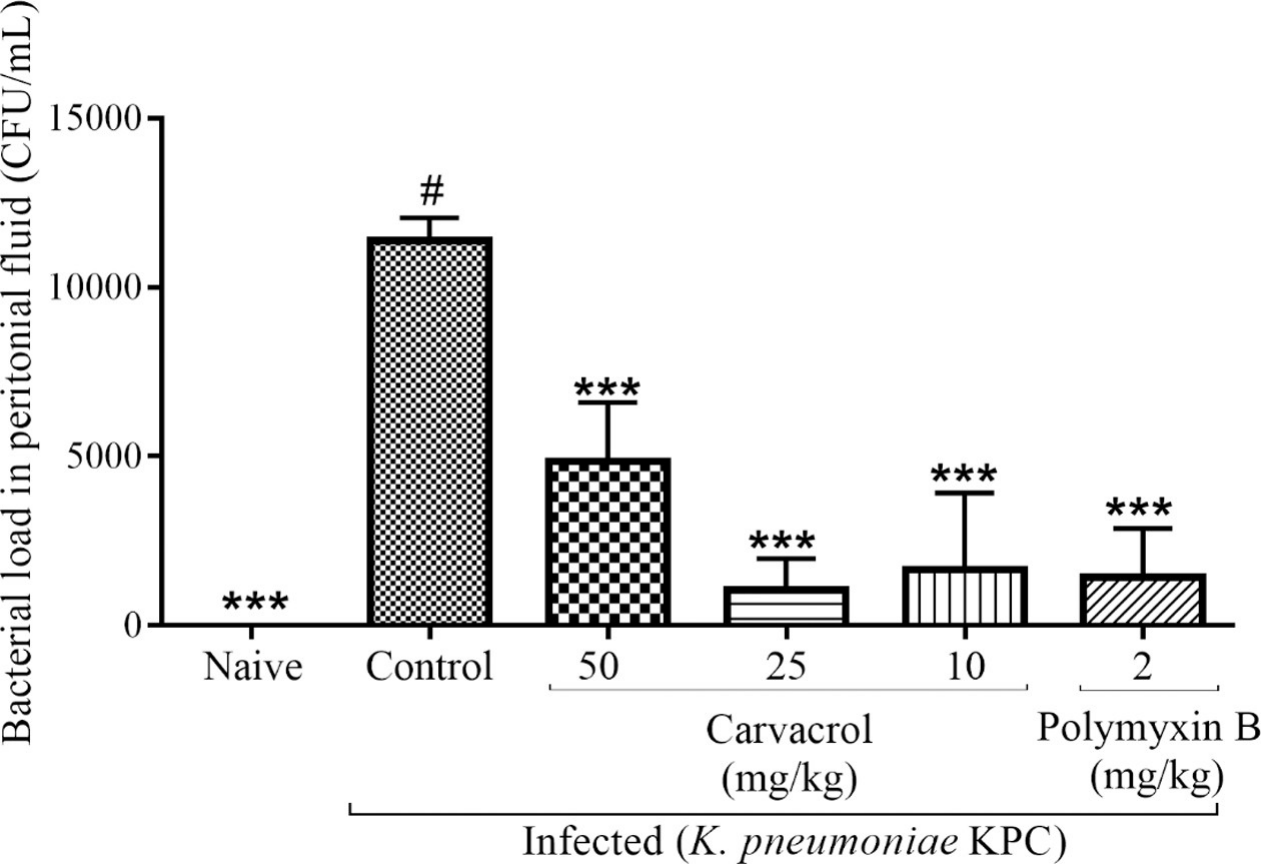
Effects of carvacrol on the number of CFUs in peritoneal lavage fluid from infected mice after 24 hours. ***P<0.001, **P<0.01 and *P<0.05 compared with the control group (#). Differences among the groups were analyzed by one-way ANOVA followed by the Newman-Keuls test.

The induction of sepsis was confirmed by bacterial culture of murine blood samples. This procedure demonstrated the presence of KPC-producing *K pneumoniae* strains in all infected mice (100%). To better characterize the difference observed for the mortality rates between the control and treated groups, we determined the number of CFUs in peritoneal lavage fluid. Bacteria was recovered from peritoneal lavage fluid of all animals, and the difference in the number of CFUs was significant for carvacrol (10, 25 and 50 mg/kg; p < 0.001) and polymyxin B (p < 0.001) (Fig 5). There was no significant difference in organ weight. Histological analysis showed no alterations in the organs.

## Discussion

*K*. *pneumoniae* is one of the most common and clinically important pathogens, causing a wide spectrum of infectious diseases [28]. The emergence and rapid spread of KPC-producing *K*. *pneumoniae* continue to pose serious threats for the treatment of healthcare-associated infections, with high mortality, especially in immunocompromised patients and neonates [29, 30]. Carbapenem-resistant *K*. *pneumoniae* isolates are particularly difficult to treat due to multifactorial resistance mechanisms that limit therapeutic options [31]. In this study, the antimicrobial activity of carvacrol against multidrug-resistant *K*. *pneumoniae* strains was assessed. For this, the antimicrobial resistance mechanisms of carbapenem-resistant *K*. *pneumoniae* isolates were analyzed, and the strains were found to acquire resistance through genes encoding carbapenemases, including *bla*_KPC-2,_ *bla*_OXA-48_, *bla*_NDM-1_, and *bla*_CTX-M-8_. The spread of carbapenemase-encoding genes among these pathogens is a cause of great concern, drastically compromising the therapeutic options available for treatment [19]. Thus, developing new therapies against these bacteria is a priority.

Carvacrol was investigated as a potential novel therapeutic agent and showed encouraging inhibitory effects against carbapenem and polymyxin-resistant *K*. *pneumoniae* strains. Carvacrol showed a low MIC and MBC value (ranging from 130 to 260 mg/L) for carbapenem and polymyxin-resistant *K*. *pneumoniae* strains. This is a promising result, as there are limited antibiotics available for treating MDR Gram-negative bacteria [32]. Carvacrol exhibited inhibitory effects (MIC = 130 mg/L) *in vitro* against KPC-producing *K*. *pneumoniae*, eradicating all bacterial cells, similar to polymyxin B and amikacin, both commercial antibiotics. Carvacrol is described as a potential antimicrobial agent against Gram-positive and Gram-negative bacteria [33]. However, to our knowledge, there is no description of the antimicrobial activity of carvacrol against MDR bacteria, as shown in our study.

The antimicrobial action of carvacrol and its time-kill curves provided evidence of its rapid action. The inhibitory effects of carvacrol could be attributed to the interactions between the structural and functional properties of the cytoplasmatic membrane, where carvacrol interacts with the lipid bilayer and aligns itself between fatty acid chains, leading to the expansion and destabilization of the cytoplasmic membrane [10, 13, 34]. Several mechanisms have been proposed to explain the antimicrobial activity of carvacrol against bacteria [10, 35–37]. However, further studies are required to elucidate the mechanisms of action and cell death caused by carvacrol in carbapenem-resistant *K*. *pneumoniae*.

*In vitro* results showed that carvacrol had similar antimicrobial activity against all isolates evaluated, indicated that the activity is not restricted to resistance genes or chromosomal polymyxin-resistant mechanisms. Thus, considering the similar results *in vitro*, antimicrobial activity of carvacrol in a murine model was evaluated with KPC-producing *K*. *pneumoniae*-induced infection. Treatment with carvacrol significantly increased the survival of infected mice compared to the control group (untreated). In addition, mice treated with carvacrol showed a significant decrease in the number of CFU in the collected peritoneal lavage, similar to the group treated with polymyxin B.

An elevated WBC count was observed in untreated mice (control group) compared to the naïve and treated groups, suggesting infectious or inflammatory processes. Moreover, carvacrol decreased the number of total leukocytes in the untreated group, similar to polymyxin B, indicating that carvacrol may be efficient in the treatment of infections since leukocytes are the first line of defense against invading pathogens. Several studies have used *in vitro* and *in vivo* assays to demonstrate that carvacrol exerts its anti-inflammatory properties by reducing the production of inflammatory mediators such as leucocytes, possibly through the induction of IL-10 release [17, 35, 36, 38, 39]. In addition, a significant increase was observed in the number of platelets in mice treated with carvacrol when compared to the untreated (control) group, suggesting that treatment with carvacrol decreased the severity of the infection. On the other hand, the group with polymyxin B did not show any differences in platelet numbers compared to the control group. This may be attributed to the fact that polymyxin B has no effect on platelet activation and can selectively inhibit platelet aggregation [40]. In the diagnosis of sepsis, the number of platelets is an important laboratory finding [41]. Platelets play a role in maintaining hemostasis, modulate innate and adaptive immune responses, and low platelet count is a marker for poor prognosis in septic patients [42]. Low platelet counts were correlated with an increased risk of infection in patients [43]. Thus, in our study, the increase in platelet count may be related to the reduced severity of the infection. The antiplatelet properties of carvacrol showed that carvacrol has a moderate antiplatelet effect, inhibiting platelet aggregation [44, 45].

In addition, carvacrol has been classified as a generally recognized safe compound and is approved for use in food items [9, 26]. Data regarding the acute and short-term *in vivo* effects in different animal species are available and suggest that carvacrol does not pose a risk to human health [36]. Nevertheless, the results of this study indicate that the use of carvacrol as a therapeutic agent can exert significant *in vitro* and *in vivo* antimicrobial effects against KPC-producing *K*. *pneumoniae*, increasing animal survival and significantly decreasing bacterial loads. However, the absence of cytokine dosage is a limitation of this study. So, further studies are needed to elucidate the role of cytokines in the antimicrobial properties of carvacrol. Also, the linear dose-response of carvacrol was not applicable to our study. Carvacrol shows a biphasic dose-response relationship, in which the low dose causes stimulation, and the dose increases an inhibition. This seems to be similar to a hormetic effect. However, the hormetic effect mechanism is extremely limited, mainly in the context of antimicrobial activities [46, 47]. Additional studies are required to elucidate the dose-response of carvacrol.

In conclusion, preliminary results in mice are hopeful and indicate that carvacrol has potential as an antimicrobial agent against KPC-producing *K*. *pneumoniae*. However, more studies of carvacrol activity and its action mechanisms in animal models are necessary to enhance our understanding and establish its efficacy.

## References

[1] Roca I, Akova M, Baquero F, Carlet J, Cavaleri M, Coenen S, et al The global threat of antimicrobial resistance: science for intervention. New Microbes New Infect. 2015;6: 22–29. 10.1016/j.nmni.2015.02.007 26029375 PMC4446399

[2] Vivas R, Barbosa AAT, Dolabela SS, Jain S. Multidrug-Resistant Bacteria and Alternative Methods to Control Them: An Overview. Microb Drug Resist. 2019;25: 890–908. 10.1089/mdr.2018.0319 30811275

[3] O’Neill J. Tackling a global health crisis: initial steps. The Review on Antimicrobial Resistance. 2 2015 Available: https://amr-review.org/sites/default/files/Report-52.15.pdf

[4] O’Neill J. Tackling drug-resistant infections globally: final report and recommendations. The Review on Antimicrobial Resistance. 5 2016 Available: https://amr-review.org/sites/default/files/160518_Final%20paper_with%20cover.pdf

[5] Marsh JW, Mustapha MM, Griffith MP, Evans DR, Ezeonwuka C, Pasculle AW, et al Evolution of Outbreak-Causing Carbapenem-Resistant *Klebsiella pneumoniae* ST258 at a Tertiary Care Hospital over 8 Years. mBio. 2019;10: e01945–19, /mbio/10/5/mBio.01945-19.atom. 10.1128/mBio.01945-19 31481386 PMC6722418

[6] Alatoom A, Elsayed H, Lawlor K, AbdelWareth L, El-Lababidi R, Cardona L, et al Comparison of antimicrobial activity between ceftolozane–tazobactam and ceftazidime–avibactam against multidrug-resistant isolates of *Escherichia coli*, *Klebsiella pneumoniae*, and *Pseudomonas aeruginosa*. Int J Infect Dis. 2017;62: 39–43. 10.1016/j.ijid.2017.06.007 28610832

[7] Lomonaco S, Crawford MA, Lascols C, Timme RE, Anderson K, Hodge DR, et al Resistome of carbapenem- and colistin-resistant *Klebsiella pneumoniae* clinical isolates. Chang Y-F, editor. PLoS ONE. 2018;13: e0198526 10.1371/journal.pone.0198526 29883490 PMC5993281

[8] WHO. Global priority list of antibiotic-resistant bacteria to guide research, discovery, and development of new antibiotics World Health Organization, 2017 https://www.who.int/medicines/publications/global-priority-list-antibiotic-resistant-bacteria/en/ (accessed 01st January 2020).

[9] Hyldgaard M, Mygind T, Meyer RL. Essential Oils in Food Preservation: Mode of Action, Synergies, and Interactions with Food Matrix Components. Front Microbiol. 2012;3 10.3389/fmicb.2012.00003 22291693 PMC3265747

[10] Nostro A, Papalia T. Antimicrobial Activity of Carvacrol: Current Progress and Future Prospectives. Recent Pat Antiinfect Drug Discov. 2012;7: 28–35. 10.2174/157489112799829684 22044355

[11] Magi G, Marini E, Facinelli B. Antimicrobial activity of essential oils and carvacrol, and synergy of carvacrol and erythromycin, against clinical, erythromycin-resistant Group A Streptococci. Front Microbiol. 2015;6 10.3389/fmicb.2015.00006 25784902 PMC4347498

[12] Man A, Santacroce L, Iacob R, Mare A, Man L. Antimicrobial Activity of Six Essential Oils Against a Group of Human Pathogens: A Comparative Study. Pathogens. 2019;8: 15 10.3390/pathogens8010015 30696051 PMC6471180

[13] Burt SA, Ojo-Fakunle VTA, Woertman J, Veldhuizen EJA. The Natural Antimicrobial Carvacrol Inhibits Quorum Sensing in *Chromobacterium violaceum* and Reduces Bacterial Biofilm Formation at Sub-Lethal Concentrations. Hayes F, editor. PLoS ONE. 2014;9: e93414 10.1371/journal.pone.0093414 24691035 PMC3972150

[14] Chueca B, Pagán R, García-Gonzalo D. Oxygenated monoterpenes citral and carvacrol cause oxidative damage in *Escherichia coli* without the involvement of tricarboxylic acid cycle and Fenton reaction. Int J Food Microbiol. 2014;189: 126–131. 10.1016/j.ijfoodmicro.2014.08.008 25146464

[15] Dati LM, Ulrich H, Real CC, Feng ZP, Sun HS, Britto LR. Carvacrol promotes neuroprotection in the mouse hemiparkinsonian model. Neuroscience. 2017;356: 176–181. 10.1016/j.neuroscience.2017.05.013 28526576

[16] Sánchez C, Aznar R, Sánchez G. The effect of carvacrol on enteric viruses. Int J Food Microbiol. 2015;192: 72–76. 10.1016/j.ijfoodmicro.2014.09.028 25310265

[17] Silva FV, Guimarães AG, Silva ERS, Sousa-Neto BP, Machado FDF, Quintans-Júnior LJ, et al Anti-Inflammatory and Anti-Ulcer Activities of Carvacrol, a Monoterpene Present in the Essential Oil of Oregano. J Med Food. 2012;15: 984–991. 10.1089/jmf.2012.0102 22892022

[18] Maciel WG, Silva KE da, Bampi JVB, Bet GM dos S, Ramos AC, Gales AC, et al Identification of São Paulo metallo-beta-lactamase-1-producing *Pseudomonas aeruginosa* in the Central-West region of Brazil: a case study. Rev Soc Bras Med Trop. 2017;50: 135–137. 10.1590/0037-8682-0284-2016 28327817

[19] Silva KE, Cayô R, Carvalhaes CG, Patussi Correia Sacchi F, Rodrigues-Costa F, Ramos da Silva AC, et al Coproduction of KPC-2 and IMP-10 in Carbapenem-Resistant *Serratia marcescens* Isolates from an Outbreak in a Brazilian Teaching Hospital. Richter SS, editor. J Clin Microbiol. 2015;53: 2324–2328. 10.1128/JCM.00727-15 25878341 PMC4473237

[20] CLSI. Performance Standards for Antimicrobial Susceptibility Testing; Twenty-Five Informational Supplement CLSI Document M100-S25. Wayne, PA: Clinical and Laboratory Standards Institute; 2015.

[21] Campana EH, Xavier DE, Petrolini FV-B, Cordeiro-Moura JR, Araujo MRE de, Gales AC. Carbapenem-resistant and cephalosporin-susceptible: a worrisome phenotype among *Pseudomonas aeruginosa* clinical isolates in Brazil. Braz J Infect Dis. 2017;21: 57–62. 10.1016/j.bjid.2016.10.008 PMC9425518 27916604

[22] Vasconcelos NG, Queiroz JHF de S, Silva KE da, Vasconcelos PC de P, Croda J, Simionatto S. Synergistic effects of *Cinnamomum cassia* L. essential oil in combination with polymyxin B against carbapenemase-producing *Klebsiella pneumoniae* and *Serratia marcescens*. Koppisch AT, editor. PLoS ONE. 2020;15: e0236505 10.1371/journal.pone.0236505 32701970 PMC7377461

[23] Ye H, Shen S, Xu J, Lin S, Yuan Y, Jones GS. Synergistic interactions of cinnamaldehyde in combination with carvacrol against food-borne bacteria. Food Control. 2013;34: 619–623. 10.1016/j.foodcont.2013.05.032

[24] Gyawali R, Zimmerman T, Aljaloud SO, Ibrahim SA. Bactericidal activity of copper-ascorbic acid mixture against *Staphylococcus aureus* spp. Food Control. 2019;111: 107062 10.1016/j.foodcont.2019.107062

[25] Toledo PVM, Aranha Junior AA, Arend LN, Ribeiro V, Zavascki AP, Tuon FF. Activity of Antimicrobial Combinations against KPC-2-Producing *Klebsiella pneumoniae* in a Rat Model and Time-Kill Assay. Antimicrob Agents Chemother. 2015;59: 4301–4304. 10.1128/AAC.00323-15 25896686 PMC4468658

[26] Andre WPP, Ribeiro WLC, Cavalcante GS, Santos JML dos, Macedo ITF, Paula HCB de, et al Comparative efficacy and toxic effects of carvacryl acetate and carvacrol on sheep gastrointestinal nematodes and mice. Vet Parasitol. 2016;218: 52–58. 10.1016/j.vetpar.2016.01.001 26872928

[27] Fukuda T, Asou E, Nogi K, Goto K. Evaluation of mouse red blood cell and platelet counting with an automated hematology analyzer. J Vet Med Sci. 2017;79: 1707–1711. 10.1292/jvms.17-0387 28845024 PMC5658564

[28] Iredell J, Brown J, Tagg K. Antibiotic resistance in Enterobacteriaceae: mechanisms and clinical implications. BMJ. 2016; h6420 10.1136/bmj.h6420 26858245

[29] da Silva KE, Maciel WG, Sacchi FPC, Carvalhaes CG, Rodrigues-Costa F, da Silva ACR, et al Risk factors for KPC-producing *Klebsiella pneumoniae*: watch out for surgery. J Med Microbiol. 2016;65: 547–553. 10.1099/jmm.0.000254 27002853

[30] Lee GC, Burgess DS. Treatment of *Klebsiella Pneumoniae* Carbapenemase (KPC) infections: a review of published case series and case reports. Ann Clin Microbiol Antimicrob. 2012;11: 32 10.1186/1476-0711-11-32 23234297 PMC3552987

[31] Zhu C, Schneider EK, Wang J, Kempe K, Wilson P, Velkov T, et al A traceless reversible polymeric colistin prodrug to combat multidrug-resistant (MDR) Gram-negative bacteria. J Control Release. 2017;259: 83–91. 10.1016/j.jconrel.2017.02.005 28174100

[32] Liu S, Ono RJ, Wu H, Teo JY, Liang ZC, Xu K, et al Highly potent antimicrobial polyionenes with rapid killing kinetics, skin biocompatibility and in vivo bactericidal activity. Biomaterials. 2017;127: 36–48. 10.1016/j.biomaterials.2017.02.027 28279920

[33] Memar MY, Raei P, Alizadeh N, Akbari Aghdam M, Kafil HS. Carvacrol and thymol: strong antimicrobial agents against resistant isolates. Rev Med Microbiol. 2017;28: 63–68. 10.1097/MRM.0000000000000100

[34] Nazzaro F, Fratianni F, De Martino L, Coppola R, De Feo V. Effect of Essential Oils on Pathogenic Bacteria. Pharmaceuticals. 2013;6: 1451–1474. 10.3390/ph6121451 24287491 PMC3873673

[35] Alagawany M, Abd El-Hack ME, Farag MR., Tiwari R, Dhama K. Biological Effects and Modes of Action of Carvacrol in Animal and Poultry Production and Health—A Review. Adv Anim Vet Sci. 2015;3: 73–84. 10.14737/journal.aavs/2015/3.2s.73.84

[36] Suntres ZE, Coccimiglio J, Alipour M. The Bioactivity and Toxicological Actions of Carvacrol. Crit Rev Food Sci Nutr. 2015;55: 304–318. 10.1080/10408398.2011.653458 24915411

[37] Tackenberg MW, Geisthövel C, Marmann A, Schuchmann HP, Kleinebudde P, Thommes M. Mechanistic study of carvacrol processing and stabilization as glassy solid solution and microcapsule. Int J Pharm. 2015;478: 597–605. 10.1016/j.ijpharm.2014.12.012 25498156

[38] Fachini-Queiroz FC, Kummer R, Estevão-Silva CF, Carvalho MD de B, Cunha JM, Grespan R, et al Effects of Thymol and Carvacrol, Constituents of *Thymus vulgaris* L. Essential Oil, on the Inflammatory Response. Evid Based Complement Alternat Med. 2012;2012: 1–10. 10.1155/2012/657026 22919415 PMC3418667

[39] Lima M da S, Quintans-Júnior LJ, de Santana WA, Martins Kaneto C, Pereira Soares MB, Villarreal CF. Anti-inflammatory effects of carvacrol: Evidence for a key role of interleukin-10. Eur J Pharmacol. 2013;699: 112–117. 10.1016/j.ejphar.2012.11.040 23220159

[40] Nishikawa M, Komada F, Uemura Y, Wada H, Deguchi K, Shirakawa S. Effect of polymyxin B on platelet aggregation induced by arachidonate. Blood & Vessel. 1988;19: 632–635. 10.2491/jjsth1970.19.632

[41] Guclu E, Durmaz Y, Karabay O. Effect of severe sepsis on platelet count and their indices. Afr H Sci. 2013;13: 333–338. 10.4314/ahs.v13i2.19 24235932 PMC3824485

[42] Assinger A. Platelets and Infection -“An Emerging Role of Platelets in Viral Infection. Front Immunol. 2014;5 10.3389/fimmu.2014.00005 25566260 PMC4270245

[43] Qu M, Liu Q, Zhao H-G, Peng J, Ni H, Hou M, et al Low platelet count as risk factor for infections in patients with primary immune thrombocytopenia: a retrospective evaluation. Ann Hematol. 2018;97: 1701–1706. 10.1007/s00277-018-3367-9 29777278 PMC6097778

[44] Enomoto S, Asano R, Iwahori Y, Narui T, Okada Y, Singab ANB, et al Hematological Studies on Black Cumin Oil from the Seeds of *Nigella sativa* L. Biol Pharm Bull. 2001;24: 307–310. 10.1248/bpb.24.307 11256491

[45] Karkabounas S, Kostoula OK, Daskalou T, Veltsistas P, Karamouzis M, Zelovitis I, et al Anticarcinogenic and antiplatelet effects of carvacrol. Exp Oncol. 2006;28: 121–5. 16837902

[46] Deng Z, Lin Z, Zou X, Yao Z, Tian D, Wang D, et al Model of Hormesis and Its Toxicity Mechanism Based on Quorum Sensing: A Case Study on the Toxicity of Sulfonamides to *Photobacterium phosphoreum*. Environ Sci Technol. 2012;46: 7746–7754. 10.1021/es203490f 22715968

[47] Morales-Fernández L, Fernández-Crehuet M, Espigares M, Moreno E, Espigares E. Study of the hormetic effect of disinfectants chlorhexidine, povidone iodine and benzalkonium chloride. Eur J Clin Microbiol Infect Dis. 2014;33: 103–109. 10.1007/s10096-013-1934-5 23893017

